# Identification and Characterization of Cytokine Genes in Breast Cancer for Predicting Clinical Outcomes

**DOI:** 10.1155/mi/8441796

**Published:** 2025-10-09

**Authors:** Zhifen Han, Yi Yuan, Min Hu, Jinxiang Wang, Bing Gao, Xiaoxi Sha

**Affiliations:** ^1^Hospital of Chengdu University of Traditional Chinese Medicine, Chengdu 610075, China; ^2^Department of Urology, Kidney and Urology Center, Pelvic Floor Disorders Center, The Seventh Affiliated Hospital, Sun Yat-sen University, Shenzhen 518107, China; ^3^Sichuan Academy of Medical Sciences and Sichuan Provincial People's Hospital, University of Electronic Science and Technology of China, Chengdu 610072, China

**Keywords:** breast cancer, cytokines, immune infiltration, immunotherapy response, risk score

## Abstract

Breast cancer is a highly heterogeneous disease with diverse clinical outcomes and treatment responses. While traditional molecular subtypes have improved patient stratification, they fail to fully capture the immune heterogeneity that influences tumor progression and therapy efficacy. Cytokines play a central role in regulating immune responses within the tumor microenvironment, yet their systematic profiling in breast cancer remains unexplored. Here we conducted a comprehensive analysis of cytokine expression across breast cancer patients using transcriptomic and clinical data. Prognostic cytokines were identified via survival analysis, and a cytokine-based molecular classification was established through consensus clustering. We identified three cytokine-driven breast cancer subtypes with distinct transcriptional profiles, immune infiltration patterns, and clinical outcomes. A cytokine-derived risk score was then developed using lasso regression and validated in external datasets to assess its predictive power for patient survival and treatment response. We then characterized the immune microenvironment of patients with different risk scores using immune infiltration analysis and single-cell RNA sequencing (RNA-seq) data. The risk score effectively stratified patients into high- and low-risk groups, with significant differences in survival outcomes. The low-risk subtype exhibited enhanced immune cell infiltration and stronger immune cell interactions, while the high-risk subtype was associated with an immunosuppressive microenvironment and a worse prognosis. Notably, patients with low-risk scores demonstrated superior responses to both immunotherapy and chemotherapy, highlighting the clinical relevance of cytokine-based classification. Our study provides the first comprehensive cytokine-based molecular subtyping of breast cancer, revealing distinct immune landscapes and prognostic implications. The cytokine-derived risk score offers a powerful tool for predicting patient survival and treatment response, with potential applications in personalized medicine and immunotherapy strategies. These findings underscore the critical role of cytokines in shaping breast cancer heterogeneity and highlight their value as biomarkers for therapeutic decision-making.

## 1. Introduction

Breast cancer is the most frequently diagnosed malignancy and one of the leading causes of cancer-related mortality in women worldwide, accounting for 313,510 new cases out of 2,001,140 total cancer diagnoses through 2024 in the United States [1, 2]. Cancer treatment has evolved significantly over the past several decades, advancing from primarily surgical approaches to incorporating radiation therapy, chemotherapy, targeted therapies, and immunotherapy. Early cancer treatments focused on surgical removal of tumors, often followed by radiation therapy to target residual cancer cells. Chemotherapy was introduced in the mid-20th century and revolutionized cancer treatment by targeting rapidly dividing cells, though it often caused severe side effects due to its lack of specificity. In recent years, significant strides have been made with targeted therapies, which focus on specific molecular alterations in cancer cells. These therapies aim to block the growth and spread of cancer by targeting the specific pathways or mutations driving the disease. Immunotherapy has also emerged as a transformative treatment option, harnessing the body's immune system to recognize and destroy cancer cells [3]. Despite significant advances in treatment strategies, including surgery, chemotherapy, radiotherapy, targeted therapy, and in recent years, immunotherapy, breast cancer remains highly heterogeneous, with considerable variability in patient prognosis and treatment response [4–6]. Traditional classification methods, such as those based on hormone receptor status, HER2 expression, and proliferation markers like Ki-67, have significantly improved treatment stratification and clinical decision-making [7, 8]. However, these classifications fail to fully capture the complexity of the tumor immune microenvironment, which plays a crucial role in shaping disease progression and therapeutic outcomes. Emerging evidence suggests that immune infiltration and immune-related molecular features influence both tumor aggressiveness and response to treatment, yet the extent to which specific immune factors contribute to breast cancer heterogeneity remains incompletely understood [9–11].

Cytokines are key mediators of immune regulation and have been recognized as critical players in tumor–immune interactions [12–15]. These signaling molecules modulate a wide range of immune processes, including the recruitment, differentiation, and activation of immune cells in the tumor microenvironment [16, 17]. Proinflammatory cytokines such as interleukins (ILs), interferons (IFNs), and tumor necrosis factors (TNFs) can enhance antitumor immunity by promoting cytotoxic T cell and natural killer (NK) cell activation, while immunosuppressive cytokines such as IL-10 and transforming growth factor-beta (TGF-β) facilitate immune evasion by suppressing T cell function and promoting regulatory T cells (Tregs) and M2 macrophages [18, 19]. Given their ability to influence immune surveillance and immune suppression, cytokines play a dual role in tumor progression and response to therapy. For instance, IFN-γ promotes the response to immunotherapy while TGF-β contributes to tumor progression [19]. However, while individual cytokines have been implicated in breast cancer pathophysiology, a comprehensive and systematic evaluation of cytokine expression patterns in breast cancer and their association with prognosis has not yet been performed.

Previous efforts to classify breast cancer based on molecular signatures have focused on various gene families, including immune-related genes, metabolic markers, and cell death related genes [20–23]. While these studies have provided important insights into breast cancer heterogeneity, they have largely overlooked the role of cytokines as a distinct molecular signature for breast cancer subtyping. Given that cytokines serve as central regulators of immune activity and tumor–immune interactions, it is crucial to systematically characterize their expression patterns in breast cancer and investigate their potential as prognostic biomarkers and predictors of therapeutic response. Understanding how cytokine-driven immune regulation shapes tumor progression could provide new insights into patient stratification and guide the development of more effective immunotherapeutic strategies.

In this study, we present a comprehensive analysis of cytokine expression in breast cancer, aiming to establish a cytokine-based classification system that reflects both tumor biology and the immune microenvironment. We systematically profile almost all cytokine expression across breast cancer subtypes, evaluate their prognostic significance, and use these molecular signatures to define distinct breast cancer subgroups. By integrating transcriptomic data with immune landscape analysis and clinical outcome prediction, we assess the relevance of cytokine-based subtyping in determining patient prognosis and treatment response, particularly in the context of immunotherapy and chemotherapy. Our findings provide new insights into the immune heterogeneity of breast cancer and highlight the potential of cytokine-based classification as a tool for improving personalized treatment strategies.

## 2. Materials and Methods

### 2.1. Data Acquisition and Preprocessing

RNA sequencing (RNA-seq) and mutation data for breast cancer patients were obtained from the The Cancer Genome Atlas (TCGA)-BRCA cohort of TCGA database. Corresponding clinical information, including survival data, molecular subtypes, and treatment responses, was also retrieved. The dataset included a total of 1224 samples, of which 1108 were from breast cancer patients and 116 were from healthy individuals. Raw gene expression counts were transformed into transcripts per million (TPM) values for normalization. An independent validation dataset was obtained from the GEO dataset (GSE15852) and preprocessed using the same normalization procedures [24].

### 2.2. Mutation Landscape Analysis

Somatic mutation profiles, including single nucleotide variations (SNVs) and copy number variations (CNVs), were analyzed to identify frequently mutated genes in breast cancer. Mutation burden and co-occurrence patterns were visualized using the maftools [25] package in R. The top 10 most frequently mutated genes were identified, and their associations with known breast cancer driver mutations were examined.

### 2.3. Cytokine Gene Selection and Expression Analysis

A curated list of cytokine genes was compiled based on a publicly available database “The Dictionary of Immune Responses to Cytokines” [26]. Differential expression analysis between tumor and normal samples was conducted using the DESeq2 package in R, with significance thresholds set at adjusted *p* < 0.05 and |log2 fold-change| > 1. A heatmap was generated to visualize cytokine expression patterns across samples by using ggplot2 [27].

### 2.4. Molecular Subtyping Based on Cytokine Expression

Consensus clustering was performed using the ConsensusClusterPlus [28] R package to classify breast cancer patients into molecular subtypes based on cytokine gene expression. Partitioning around medoids (PAMs) was used as the clustering algorithm, with Pearson correlation as the distance metric. The clustering was run for a maximum of 10 clusters (max*K* = 10), with 1000 iterations (reps = 1000) to ensure robust and stable classification. In each iteration, 80% of the samples (*p*Item = 0.8) were randomly selected, while all genes (*p*Feature = 1) were included to maintain consistency in feature selection. The optimal number of clusters was determined by evaluating the consensus cumulative distribution function (CDF) and the delta area plot, which measure cluster stability across iterations. Principal component analysis (PCA) was performed using the FactoMineR and factoextra [29, 30], with the first two principal components visualized using fviz_pca_ind().

### 2.5. Immune Microenvironment Characterization

The immune composition of different cytokine-based subtypes was analyzed using CIBERSORT [31] integrated by IOBR [32], which estimates the relative proportions of immune cells from bulk RNA-seq data. Immune cell infiltration levels were further validated using ESTIMATE [33] and TIMER [34] scores. Associations between subtypes and immune infiltration were examined using Kruskal–Wallis tests.

### 2.6. Differential Gene Expression (DEG) and Pathway Enrichment Analysis

To identify key transcriptional differences among subtypes, DEG analysis was performed using DESeq2 for pairwise comparisons. Genes with adjusted *p* < 0.01 and |log2 fold-change| > 1.5 were considered significant. Gene Ontology (GO) and Kyoto Encyclopedia of Genes and Genomes (KEGG) pathway enrichment analyses were conducted using the clusterProfiler [35] package, visualized by enrichplot. Chrod diagram was made by GOplot [36] package based on GO result.

### 2.7. Development of a Cytokine-Derived Risk Score

Lasso regression was employed to construct a prognostic risk model based on cytokine expression. The glmnet [37] R package was used to select cytokines with the strongest association with overall survival. The risk score was calculated using a weighted linear combination of selected cytokines: risk score = −0.451238 × TSLP−0.8455088 × IL21 + 0.27956222 × IL27−0.07326518 × IL12B + 0.44877397 × IL10−0.02498679 × IFNG. Patients were stratified into high-risk and low-risk groups using the median risk score as the cutoff. Kaplan–Meier survival analysis was performed using the survival [38] package, and the predictive performance of the risk model was evaluated using receiver operating characteristic (ROC) curves and the area under the curve (AUC). An independent dataset was used for external validation.

### 2.8. Single-Cell RNA-Seq Analysis

Single-cell RNA-seq data were obtained from a single-cell sequencing work comparing the tumor immune microenvironment between clinal responder and nonresponder of BRCA patients [39]. Single-cell transcriptomes were analyzed using the Seurat [40] package, and risk scores were computed for each patient by averaging normalized gene expression value of cells. Uniform manifold approximation and projection (UMAP) was used for visualization of immune cell clusters. Cell–cell communication networks were inferred using CellChat [41], and interaction strengths were compared between risk groups.

### 2.9. Association Between Risk Score and Treatment Response

To evaluate the predictive value of the cytokine-derived risk score for treatment response, we analyzed real-world patient outcomes in cohorts receiving anti-PD-L1 immunotherapy and chemotherapy. Differences in immune scores between responders and nonresponders were assessed using Wilcoxon rank-sum tests. Correlation analysis was performed to identify immune-related pathways associated with treatment response.

### 2.10. Statistical Analysis

All statistical analyses were conducted using R (v4.10.2) and Python (v3.8). Survival differences between groups were evaluated using Kaplan–Meier analysis and log-rank tests. Correlations between cytokine expression and immune cell infiltration were assessed using Spearman correlation coefficients. A *p*-value < 0.05 was considered statistically significant.

## 3. Results

### 3.1. Mutational Landscape of Breast Cancer

**Figure 1:**
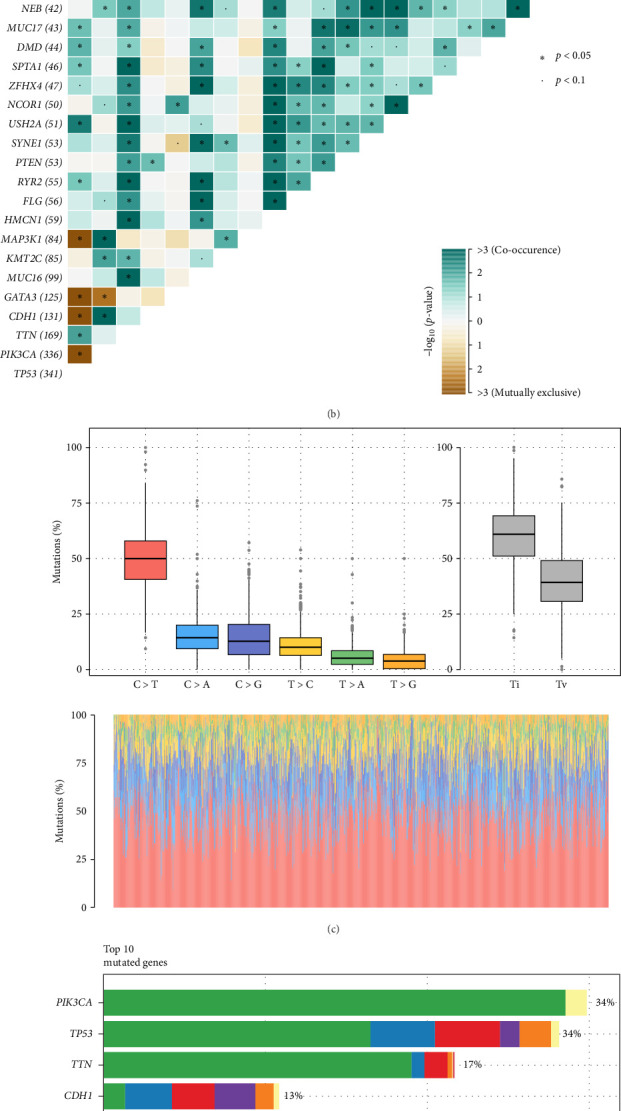
Mutation landscape of breast cancer in the TCGA cohort. (A) Copy number variation (CNV) profile across the genome, highlighting recurrent amplifications (red) and deletions (blue) in breast cancer. (B) Co-occurrence and mutual exclusivity of frequently mutated genes in breast cancer, with statistically significant comutation patterns indicated (*p* < 0.05). (C) Mutation spectrum analysis displaying the frequency of different single nucleotide substitutions. (D) Top 10 most frequently mutated genes in breast cancer, with bar length and color distribution indicating mutation type and prevalence.

We first characterized the genomic landscape in breast cancer. We analyzed somatic mutations and CNVs in the TCGA breast cancer cohort. Across the genome, we observed recurrent amplifications and deletions, with notable gains in oncogenic regions such as 8q24 and losses in tumor suppressor loci like 16q. These structural alterations likely contribute to breast cancer progression by activating oncogenes or inactivating tumor suppressors (Figure 1A). Beyond CNV events, we explored the mutational patterns of key driver genes. TP53 and PIK3CA emerged as the most frequently mutated genes, each occurring in 34% of the samples (Figure 1D). These frequencies are consistent with prior studies, such as the TCGA pan-cancer analyses, where TP53 mutations are commonly observed in approximately 30%–40% of cancer samples, and PIK3CA mutations are frequently reported in breast cancer at similar frequencies [42]. Other recurrently mutated genes included *CDH1*, *GATA3*, *MAP3K1*, and *MUC16*, which are known to influence tumor behavior through pathways regulating cell adhesion, transcriptional control, and immune evasion. Further investigation of comutation patterns revealed significant interactions between certain gene alterations. *TP53* mutations tended to co-occur with mutations in *NF1* and *SPTA1*, while *PIK3CA* mutations frequently coexisted with *GATA3* and *MAP3K1* mutations, suggesting potential cooperative roles in tumor development (Figure 1B). In contrast, mutually exclusive relationships were observed between *TP53* and *CDH1*, consistent with their distinct molecular roles in different breast cancer subtypes. The mutational spectrum analysis showed a predominance of C > T transitions, a hallmark of spontaneous deamination, along with a substantial contribution of transversions (Figure 1C). This pattern aligns with previously reported mutational processes in breast cancer, highlighting the influence of both endogenous and exogenous mutagenic factors. Together, these findings provide a comprehensive view of the genomic landscape of breast cancer, laying the foundation for further exploration of molecular subtypes and their distinct mutation-driven characteristics.

#### 3.1.1. Dysregulation of Cytokine Expression in Breast Cancer

**Figure 2:**
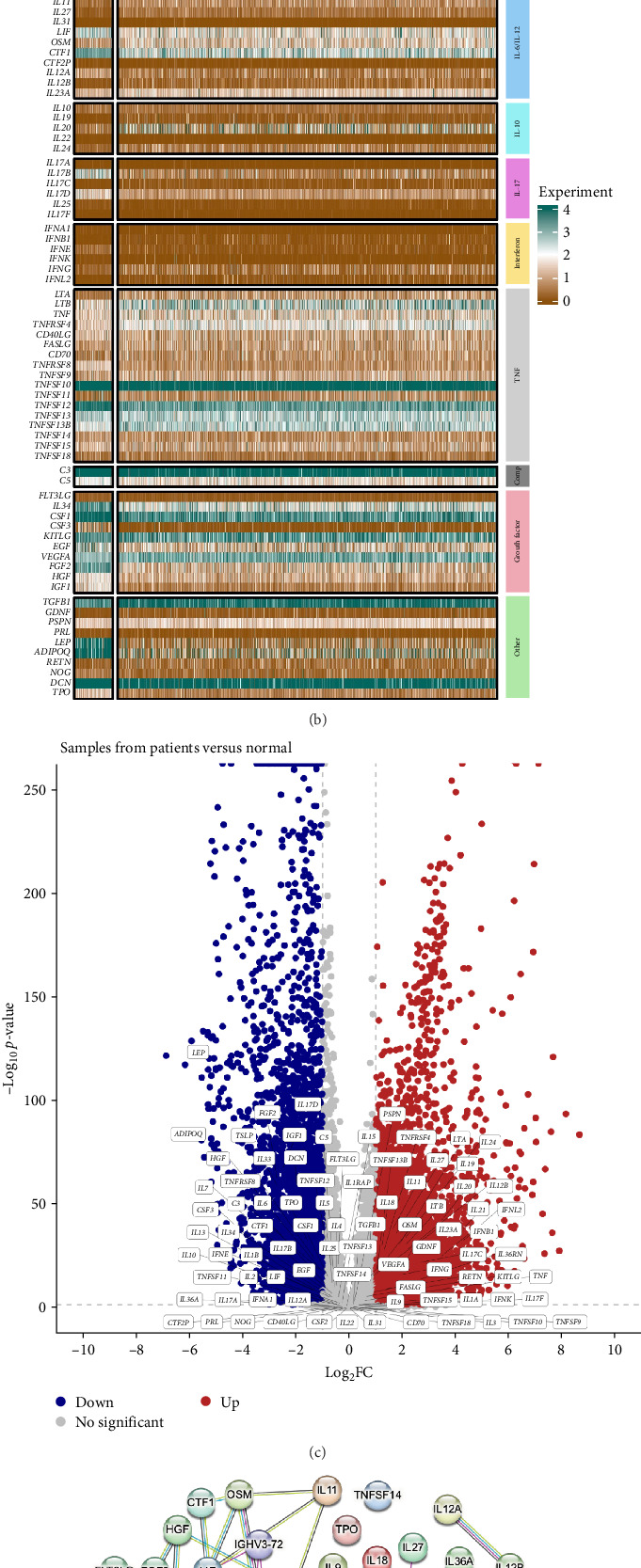
Cytokine expression profiling in breast cancer. (A) Boxplots comparing cytokine expression levels between tumor and normal breast tissues. (B) Heatmap showing the expression levels of classified cytokines in healthy individuals (Nor) and breast cancer patients (patient), with color gradients representing expression from low (brown) to high (green). (C) Volcano plot identifying differentially expressed cytokines between tumor and normal tissues (*p* < 0.05, log2FC threshold applied). (D) Protein–protein interaction network of differentially expressed cytokines, highlighting key regulatory hubs in cytokine signaling.

Given the critical role of cytokines in tumor immunity, we systematically analyzed their expression profiles in breast cancer using a curated cytokine list [26]. Overall expression patterns revealed significant differences between tumor and normal tissues (Figure 2A). While certain cytokines exhibited elevated expression in tumors, others were downregulated, indicating potential immunosuppressive or protumorigenic shifts. A heatmap representation further confirmed these trends, with distinct expression clusters emerging across patient samples (Figure 2B). Notably, cytokines associated with proinflammatory responses, such as *IL6*, *TNF*, and *IL1B*, showed marked upregulation in tumors, while several homeostatic cytokines, including *IL10* and *TGFB1*, were also altered, suggesting a complex interplay between inflammatory and regulatory signals. To identify cytokines with significant differential expression, we performed a comparative analysis between tumor and normal samples (Figure 2C). The volcano plot highlighted key cytokines that were either upregulated (e.g., *CCL2*, *IL1B*, and *CSF2*) or downregulated (e.g., *IL7* and *FLT3LG*), reflecting distinct immunological microenvironments. These findings suggest that breast cancer progression involves shifts in cytokine-mediated signaling, potentially shaping immune evasion and tumor-promoting inflammation. Further investigation of cytokine–cytokine interactions using a network-based approach revealed intricate functional associations among differentially expressed cytokines (Figure 2D). Highly interconnected nodes, including *IL6*, *IL1B*, and *CCL5*, suggest key regulatory hubs orchestrating immune responses in the tumor microenvironment. The presence of multiple interaction clusters underscores the complexity of cytokine signaling in breast cancer, reinforcing the notion that both pro- and anti-inflammatory cytokines contribute to tumor progression. Collectively, these findings provide a comprehensive view of cytokine dysregulation in breast cancer, highlighting potential targets for immunomodulatory interventions.

### 3.2. Identification of Cytokine-Based Molecular Subtypes in Breast Cancer

**Figure 3:**
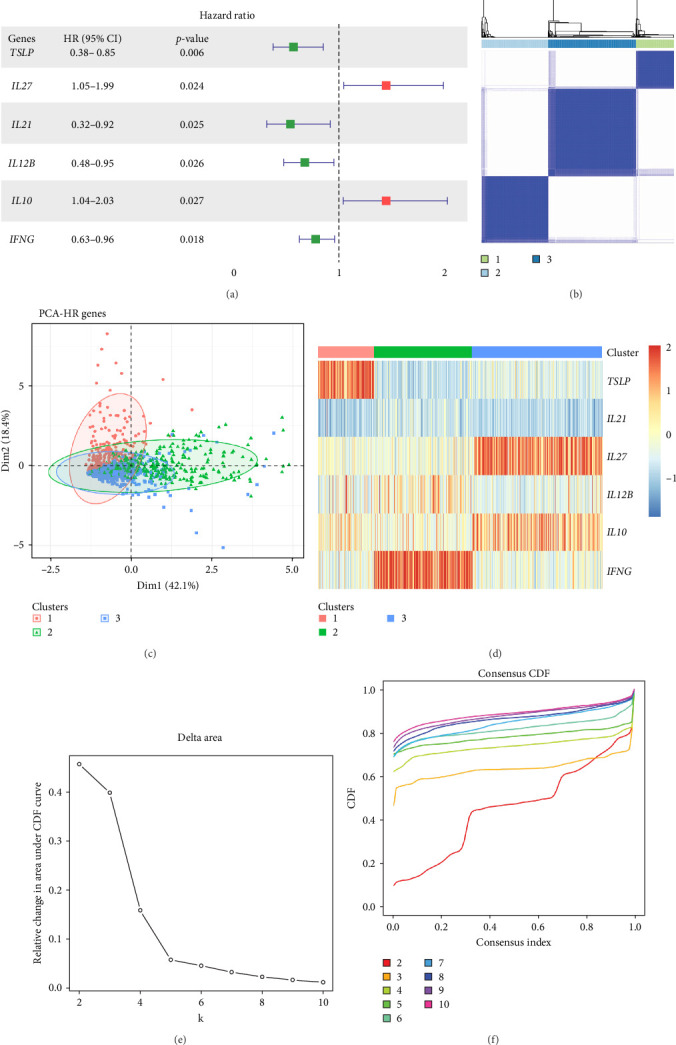
Cytokine-based molecular subtyping of breast cancer. (A) Forest plot displaying the hazard ratios of survival-associated cytokines. (B) Consensus clustering matrix supporting the classification of breast cancer patients into three molecular subtypes based on cytokine expression. (C) Principal component analysis (PCA) confirming the distinct clustering of patients by subtype. (D) Heatmap showing the expression of survival-associated cytokines across the three subtypes. (E) Delta area plot illustrating the optimal number of clusters. The relative gain in cluster stability decreased notably beyond three clusters. (F) Consensus cumulative distribution function (CDF) plot validating the stability of clustering. The CDF curve at *k* = 3 showed stable convergence, indicating robust clustering.

To explore the prognostic significance of cytokines in breast cancer, we systematically analyzed the hazard ratios (HRs) of all cytokines and identified a subset of key cytokines with significant associations with patient survival (Figure 3A). Among them, *TSLP*, *IL21*, *IL12B*, and *IFNG* were associated with a favorable prognosis, whereas *IL27* and *IL10* were linked to poorer outcomes. These results suggest that cytokine expression patterns could have significant prognostic relevance in breast cancer. To further investigate whether these cytokines could stratify breast cancer patients into distinct molecular subtypes, we performed consensus clustering based on their expression profiles. The consensus matrix (Figure 3B) demonstrated a clear partitioning of the cohort into three stable clusters. PCA (Figure 3C) further confirmed this classification, with distinct clustering patterns observed along the major principal components, indicating that cytokine expression signatures define biologically meaningful subtypes. Cytokine expression across these three clusters (Figure 3D) revealed distinct expression patterns, with *TSLP*, *IL21*, and *IL12B* being highly expressed in one subtype, while *IL27* and *IL10* were enriched in another. This suggests that these subtypes may be associated with different immune microenvironmental states. To determine the optimal number of clusters, we evaluated the relative change in the area under the CDF curve across different *k*-values (Figure 3E), which indicated that *k = 3* was the most stable clustering solution. The consensus CDF plot (Figure 3F) further supported the robustness of the three-subtype classification. Together, these findings suggest that cytokine expression patterns define three distinct molecular subtypes of breast cancer, which may have implications for patient prognosis and response to immune-targeted therapies.

### 3.3. Immune Microenvironment Characterization of Cytokine-Based Breast Cancer Subtypes

**Figure 4:**
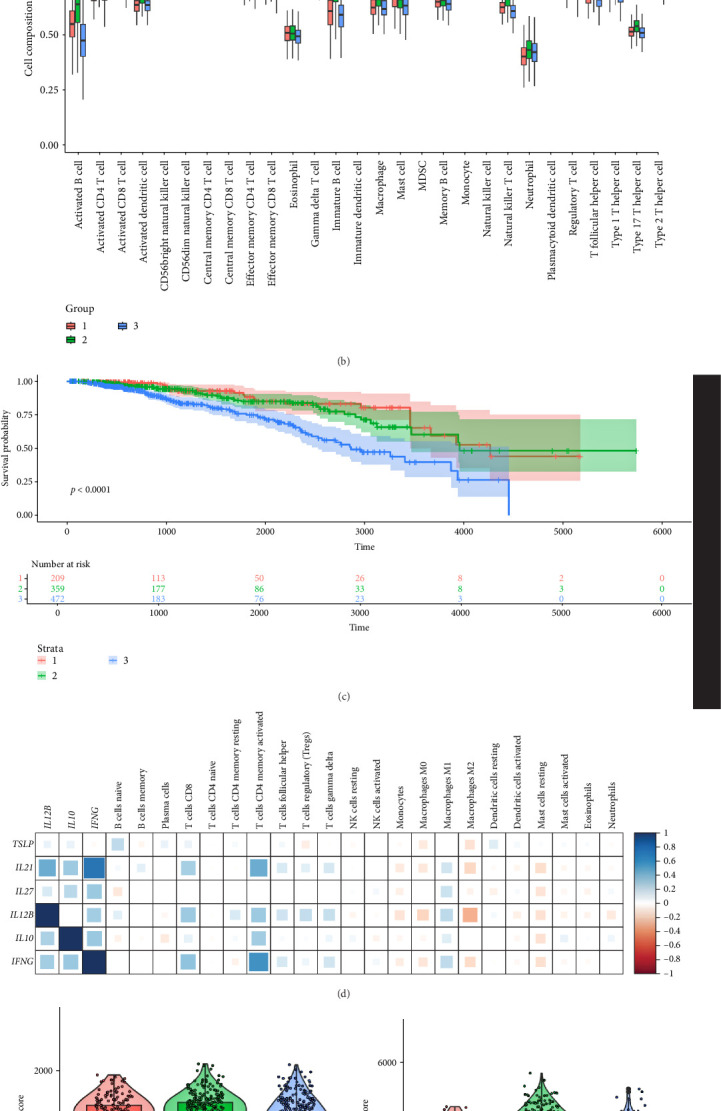
Immune microenvironment differences among cytokine-based breast cancer subtypes. (A) Heatmap showing the relative abundance of immune cell types across the three subtypes based on CIBERSORT analysis. (B) Boxplots comparing immune cell composition between subtypes, revealing significant differences in immune infiltration (*p* < 0.05). (C) Kaplan–Meier survival curve showing significant differences in overall survival between the three subtypes (*p* < 0.0001). (D) Correlation heatmap depicting associations between key cytokines and immune cell infiltration levels. (E–G) Comparisons of stromal score, ESTIMATE score, and immune score between the three subtypes, indicating significant differences in tumor microenvironment composition.

To investigate the immune landscape associated with the three cytokine-based molecular subtypes, we performed CIBERSORT analysis to estimate the composition of immune cells in each subtype. The analysis (Figure 4A) reveals distinct immune infiltration patterns among the subtypes, with certain cell populations showing enrichment in specific groups. Notably, subtype 1 exhibited a higher presence of activated CD4 + T cells, NK cells, and dendritic cells, whereas subtype 3 was characterized by a stronger presence of macrophages and Tregs, suggesting a more immunosuppressive environment. A more detailed comparison of immune cell compositions across subtypes (Figure 4B) further confirmed significant differences in various immune populations. Subtype 1 showed higher levels of effector T cells and antigen-presenting cells, indicative of an active immune response, whereas subtype 3 exhibited an increased proportion of monocytes, M2 macrophages, and Tregs, which are often associated with immune evasion and tumor progression. Survival analysis (Figure 4C) demonstrated a significant difference in prognosis among the three subtypes. Patients classified in subtype 1, characterized by a more active immune environment, had a significantly better prognosis, while those in subtype 3, associated with an immunosuppressive microenvironment, had the poorest overall survival. To further explore the relationship between immune infiltration and tumor microenvironment composition, we examined key immune scores, including the stromal score, ESTIMATE score, and immune score (Figure 4E–G). Subtype 3 exhibited significantly higher stromal and immune scores compared to the other subtypes, suggesting increased immune infiltration and stromal activation, which are often linked to a more complex and potentially therapy-resistant microenvironment. Correlation analysis between key cytokines and immune cell abundance (Figure 4D) further highlighted potential interactions shaping the tumor immune landscape. Proinflammatory cytokines such as *IL1B* and *IFNG* were positively correlated with effector T cell abundance, while *IL10*, known for its immunosuppressive role, showed strong associations with Treg and M2 macrophage infiltration. Overall, these findings illustrate the immunological heterogeneity of the cytokine-defined breast cancer subtypes, providing insights into their distinct tumor–immune interactions and potential implications for immunotherapy responsiveness.

### 3.4. Cytokine-Based Subtypes Exhibit Distinct Transcriptional Programs and Immune Signatures

**Figure 5:**
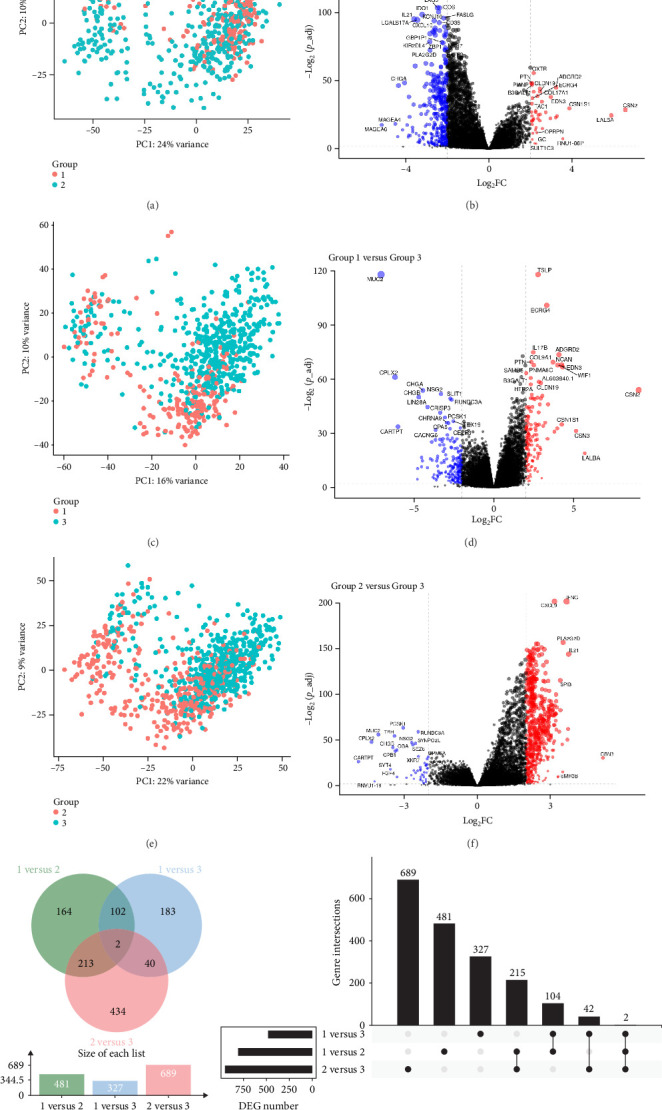
Differential gene expression analysis across cytokine-based breast cancer subtypes. (A,C,E) Principal component analysis (PCA) illustrating distinct clustering of differentially expressed genes (DEGs) between subtypes. (B,D,F) Volcano plots highlighting significantly upregulated and downregulated genes in pairwise subtype comparisons (*p* < 0.05, log2FC threshold applied). (G) Venn diagram summarizing the overlap of DEGs across subtypes. (H) UpSet plot quantifying the number of shared and unique DEGs among subtype pairs.

To further characterize the molecular differences among the three cytokine-defined breast cancer subtypes, we performed DEG analysis to identify key transcriptional changes distinguishing each group. PCA revealed distinct clustering of gene expression profiles between subtypes (Figure 5A,C,E), indicating significant transcriptomic heterogeneity among the groups. Pairwise DEG comparisons were conducted to identify significantly upregulated and downregulated genes between subtypes (Figure 5B,D,F). The comparison between subtype 1 and subtype 2 highlighted an enrichment of immune-related genes, such as *IFNG*, *CXCL9*, and *GBP5*, suggesting that subtype 1 may be associated with a stronger immune response (Figure 5B). In contrast, subtype 3 displayed upregulation of *TSLP*, *IL17B*, and *ECRG4*, indicating potential immunosuppressive and tumor-promoting mechanisms (Figure 5D). The subtype 2 versus subtype 3 comparison further revealed distinct gene expression profiles, with *PLAG2G2D*, *IL21*, and *SPP1* being upregulated in subtype 2, while *MUC16* and *PCSK1* were enriched in subtype 3 (Figure 5F). To summarize the shared and unique DEGs across comparisons, we generated a Venn diagram (Figure 5G) displaying the overlap of DEGs among the three subtypes. While a small subset of genes (two genes) were differentially expressed across all comparisons, each pairwise comparison also revealed unique gene signatures, emphasizing the molecular diversity between subtypes. The UpSet plot (Figure 5H) further quantified these shared and distinct DEGs, illustrating the largest number of unique genes distinguishing subtype 1 from subtype 3. Overall, these findings reinforce the distinct transcriptional landscapes of the three cytokine-based breast cancer subtypes, with key immune-related and tumor-associated genes driving their molecular identities. These results provide further insights into the potential biological mechanisms underlying breast cancer heterogeneity.

### 3.5. A Cytokine-Based Risk Model Predicts Breast Cancer Prognosis

**Figure 6:**
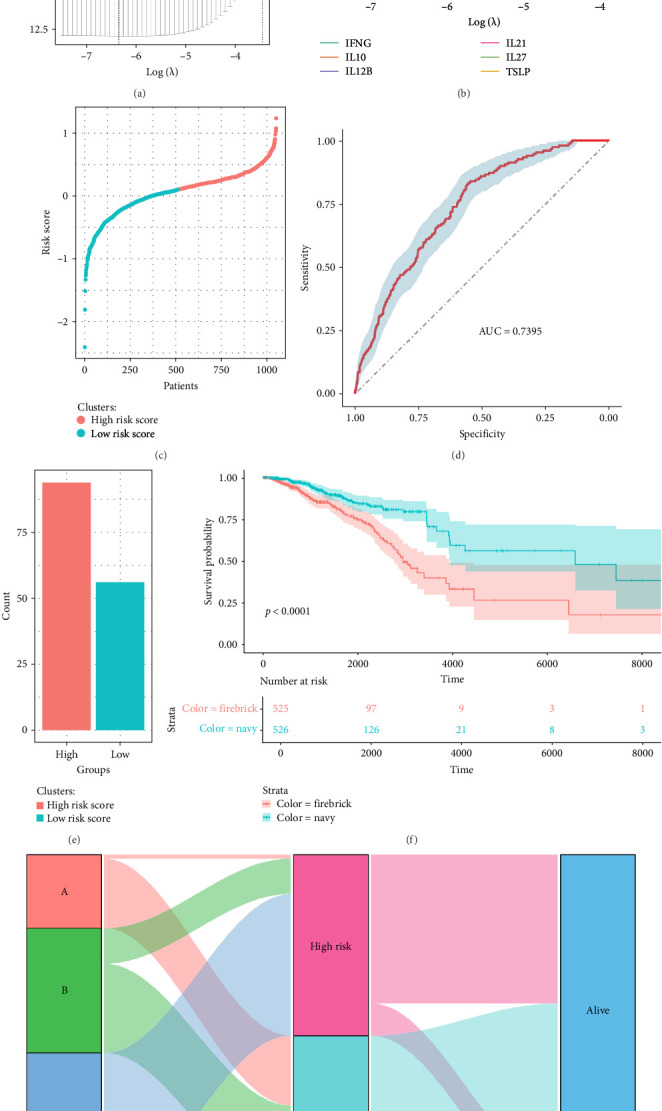
Cytokine-derived risk score predicts breast cancer prognosis. (A) Lasso regression model selection plot identifying the optimal lambda value for risk score computation. (B) Coefficients of selected cytokines contributing to the risk score. (C) Distribution of risk scores among breast cancer patients, stratifying them into high- and low-risk groups. (D) Receiver operating characteristic (ROC) curve assessing the predictive accuracy of the risk score model (AUC = 0.7395). (E) Distribution of high- and low-risk patients. (F) Kaplan–Meier survival analysis demonstrating a significant survival difference between risk groups (*p* < 0.0001). (G) Sankey plot illustrating the transition from cytokine-based subtypes to risk groups and survival outcomes.

To assess the prognostic value of cytokine expression, we constructed a lasso regression model to develop a cytokine-derived risk score. The optimal lambda value was selected based on the partial likelihood deviance (Figure 6A), and the coefficient paths of cytokines contributing to the model were identified (Figure 6B). The final risk score was computed as: risk score = −0.451238 × TSLP−0.8455088 × IL21 + 0.27956222 × IL27−0.07326518 × IL12B + 0.44877397 × IL10−0.02498679 ×IFNG. This risk score was then used to stratify patients into high-risk and low-risk groups, with a clear separation observed in the distribution of scores across patients (Figure 6C). To evaluate the predictive power of the model, we validated it using an external dataset, demonstrating an area under the ROC curve (AUC = 0.7395, Figure 6D). This indicated a robust predictive performance, which is comparable with established prognostic models [43, 44]. The distribution of patients across high- and low-risk groups (Figure 6E) showed a significant association with survival outcomes, where high-risk patients had notably worse prognosis. Kaplan–Meier survival analysis (Figure 6F) revealed a significant survival difference between the two groups (*p* < 0.0001), with high-risk patients experiencing poorer overall survival compared to low-risk patients. Finally, a Sankey plot (Figure 6G) illustrates the transition from cytokine-based molecular subtypes to risk classification and survival status, reinforcing the relevance of cytokine expression in defining breast cancer prognosis. Together, these results establish a cytokine-based risk model as a powerful tool for predicting breast cancer outcomes, offering potential clinical applications for risk stratification and personalized treatment strategies.

### 3.6. High-Risk and Low-Risk Patients Exhibit Distinct Immune-Related Transcriptional Profiles

**Figure 7:**
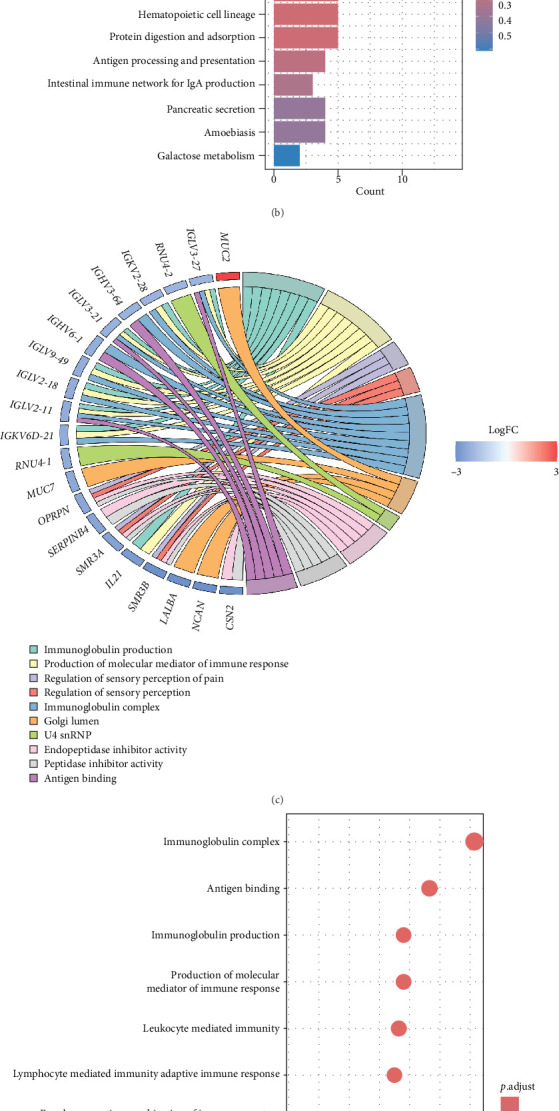
High-risk and low-risk patients exhibit distinct immune-related transcriptional programs. (A) Volcano plot identifying differentially expressed genes (DEGs) between high- and low-risk groups, with immune-related genes highlighted. (B) KEGG pathway enrichment analysis demonstrating significant enrichment in immune-related pathways. (C) Chord diagram illustrating key gene-pathway interactions. (D) Gene Ontology (GO) enrichment analysis of DEGs, highlighting pathways involved in antigen presentation, immunoglobulin production, and adaptive immunity.

To evaluate whether the cytokine-derived risk score truly reflects differences in prognosis and immune heterogeneity, we performed DEG analysis between high-risk and low-risk patients. The volcano plot (Figure 7A) highlights significantly upregulated and downregulated genes in the high-risk group, revealing a distinct transcriptional landscape associated with poor prognosis. Pathway enrichment analysis of the DEGs confirmed a strong immune-related signature. KEGG pathway analysis (Figure 7B) showed significant enrichment in immune-related pathways, including primary immunodeficiency, cytokine–cytokine receptor interactions, and B cell receptor signaling, suggesting that immune dysregulation plays a central role in driving the poor prognosis of high-risk patients. GO enrichment analysis further validated these findings (Figure 7D), with DEGs predominantly involved in antigen binding, immunoglobulin complex formation, and leukocyte-mediated immunity. These results indicate that high-risk patients exhibit substantial alterations in immune processes, which may contribute to tumor progression and immune evasion. A chord diagram (Figure 7C) illustrates the relationships between key DEGs and their enriched immune functions, providing a global view of the immune interactions distinguishing high- and low-risk patients. Many of the identified genes are involved in immunoglobulin production, antigen processing, and cytokine regulation, reinforcing the hypothesis that variations in cytokine signaling impact breast cancer prognosis. These findings demonstrate that the cytokine-based risk score effectively captures not only survival differences but also underlying immune heterogeneity, supporting its potential utility in guiding immunotherapy strategies for breast cancer patients.

### 3.7. Low-Risk Score Patients Exhibit Greater Immune Infiltration and Stronger Immune Cell Interactions

**Figure 8:**
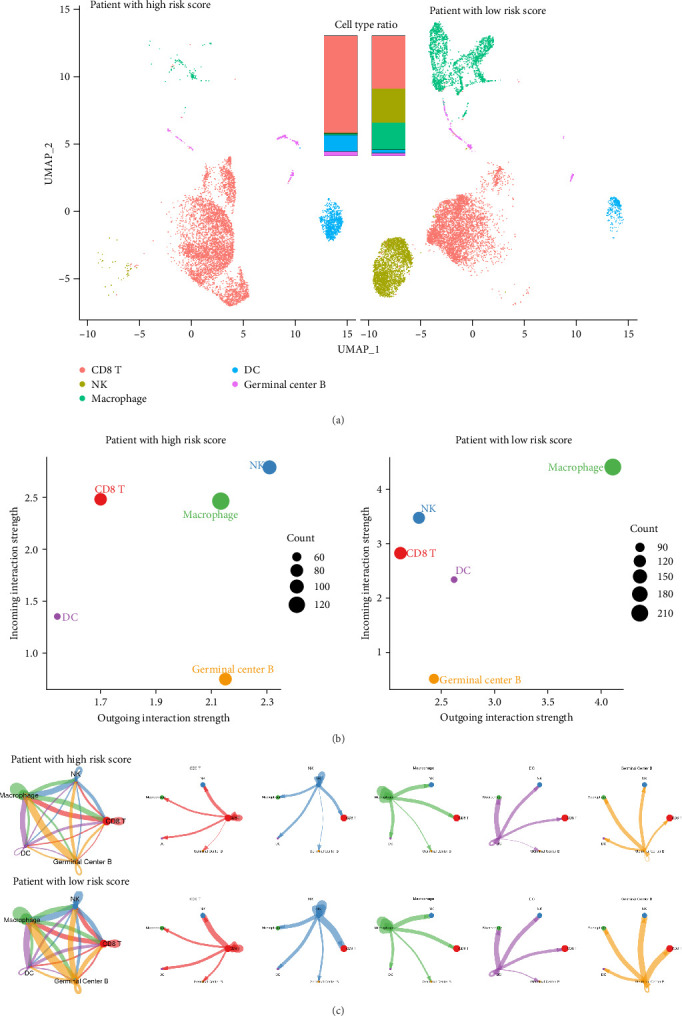
Low-risk score patients exhibit greater immune infiltration and stronger immune cell interactions. (A) UMAP projection of immune cell populations, revealing significantly greater immune infiltration in low-risk patients. (B) CellChat analysis comparing incoming and outgoing interaction strengths among major immune cell types, showing stronger immune communication in low-risk patients. (C) Cell–cell interaction network illustrating enhanced immune interactions in low-risk patients, particularly between CD8+ T cells, NK cells, macrophages, and germinal center B cells.

To further validate the biological relevance of our risk score model, we leveraged publicly available single-cell RNA-seq data from breast cancer patients [37]. We computed risk scores for individual patients and stratified them into high- and low-risk groups, revealing significant differences in immune composition and cell–cell interactions. The UMAP projection of immune cell populations (Figure 8A) demonstrated that low-risk patients exhibited significantly greater immune infiltration compared to high-risk patients. The low-risk group showed higher proportions of CD8 + T cells, NK cells, macrophages, and dendritic cells, all of which play crucial roles in antitumor immunity. The cell type ratio comparison further confirmed an immune-rich tumor microenvironment in low-risk patients, whereas high-risk patients displayed a more immunosuppressive landscape. We next examined immune cell interactions using CellChat analysis, which revealed that immune communication was notably stronger in low-risk patients. A comparison of incoming and outgoing interaction strengths among major immune cell types (Figure 8B) showed that CD8+ T cells, NK cells, and macrophages had significantly greater interaction strengths in low-risk patients, suggesting enhanced immune coordination. The overall immune network (Figure 8C) reinforced this trend, with low-risk patients displaying highly interconnected immune cell communication. CD8+ T cells, NK cells, macrophages, and germinal center B cells exhibited extensive cross-talk, indicative of an active and responsive immune environment. In contrast, high-risk patients showed a weaker immune network, suggesting impaired immune function and a potentially more immunosuppressive microenvironment. These results provide strong evidence that low-risk score patients exhibit both higher immune infiltration and stronger immune cell interactions, which may contribute to their better prognosis and greater likelihood of responding to immunotherapy. This further supports the utility of our cytokine-based risk model in predicting immune heterogeneity and guiding treatment strategies.

#### 3.7.1. Low-Risk Score Patients Respond Better to Both Immunotherapy and Chemotherapy

**Figure 9:**
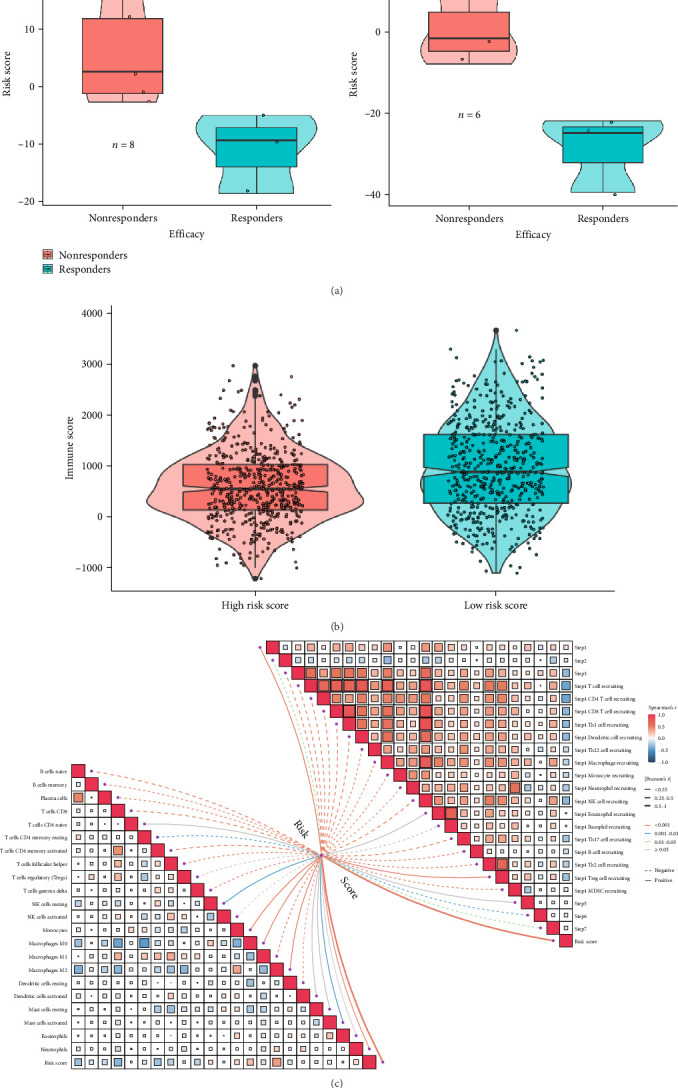
Low-risk patients exhibit better responses to both immunotherapy and chemotherapy. (A) Boxplots comparing immune scores between responders and nonresponders in patients receiving anti-PD-L1 therapy and chemotherapy, showing significantly higher immune scores in responders (*p* < 0.05). (B) Comparison of immune scores between high- and low-risk patients, confirming stronger immune activation in the low-risk group (*p* < 0.0001). (C) Correlation matrix illustrating associations between risk scores and immune cell recruitment pathways, with positive correlations observed between low-risk scores and CD8+ T cell, dendritic cell, and macrophage recruitment pathways.

To further assess the clinical relevance of our cytokine-based risk score, we analyzed real-world treatment response data from single-cell RNA-seq datasets containing patient outcomes to anti-PD-L1 immunotherapy and chemotherapy. This allowed us to determine whether risk stratification correlates with actual treatment efficacy. Patients in the low-risk group demonstrated significantly better responses to both immunotherapy and chemotherapy compared to high-risk patients. In the anti-PD-L1-treated cohort (Figure 9A), immune responders had significantly lower risk scores, indicating that tumors with stronger immune infiltration are more likely to benefit from immune checkpoint blockade. Similarly, in the chemotherapy-treated cohort (Figure 9A), low-risk patients exhibited better treatment responses, reinforcing the idea that immune-enriched tumors are more sensitive to traditional chemotherapy. Comparison of immune scores across risk groups (Figure 9B) further confirmed that low-risk patients had significantly higher immune scores, reflecting a more active and immunologically “hot” tumor microenvironment. These findings suggest that tumors classified as high-risk may exhibit immune exclusion or suppression, contributing to their resistance to therapy. To explore the underlying mechanisms linking risk score to treatment response, we performed a correlation analysis between risk score and immune cell recruitment pathways (Figure 9C). Low-risk patients exhibited stronger correlations with pathways involved in CD8+ T cell, dendritic cell, and macrophage recruitment, all of which are crucial for effective antitumor immunity. In contrast, high-risk patients were associated with pathways linked to immune suppression, such as Tregs and myeloid-derived suppressor cells (MDSCs), which are known to dampen immune responses. These findings provide strong evidence that the cytokine-based risk score not only reflects tumor immune heterogeneity but also serves as a predictor of clinical response to both immunotherapy and chemotherapy. Low-risk patients, characterized by a more active immune microenvironment, are significantly more likely to benefit from treatment, while high-risk patients may require additional strategies to overcome immune resistance.

## 4. Discussion

In this study, we performed a comprehensive analysis of cytokine gene expression in breast cancer, revealing distinct molecular subtypes with unique immune landscapes and prognostic implications. By systematically profiling cytokines across breast cancer patients, we identified three cytokine-based subtypes with differing immune infiltration patterns, survival outcomes, and therapeutic responses. Our findings provide novel insights into the role of cytokine signaling in shaping the tumor microenvironment and highlight the potential of cytokine-based classification as a valuable tool for breast cancer stratification.

One of the key findings of our study is the strong association between cytokine-driven subtypes and immune infiltration. The low-risk subtype exhibited significantly higher immune cell infiltration, particularly of CD8 + T cells, NK cells, and antigen-presenting cells, similar to immune subtypes identified in previous studies [43], suggesting a more active antitumor immune response. In contrast, the high-risk subtype was characterized by an immunosuppressive microenvironment, with an increased presence of Tregs and M2 macrophages. Additionally, macrophages, particularly tumor-associated macrophages (TAMs), play a crucial role in breast cancer progression. Recent research has demonstrated how imaging techniques combined with genetic data can provide valuable insights into the immune landscape of breast cancer, aiding in patient stratification and treatment planning [45].

Furthermore, we developed a cytokine-based risk score that effectively stratifies patients into high- and low-risk groups with significant differences in overall survival. This risk score demonstrated robust predictive power in external validation datasets, underscoring its clinical applicability. Importantly, our analysis revealed that low-risk patients exhibited superior responses to both immunotherapy and chemotherapy, suggesting that cytokine signaling plays a crucial role in determining treatment efficacy. This finding has important clinical implications, as it suggests that cytokine profiles could be used to guide therapeutic decision-making and identify patients who are more likely to benefit from immune checkpoint inhibitors or conventional chemotherapy.

Our study also provides mechanistic insights into the functional roles of cytokines in breast cancer progression. Gene enrichment analyses of differentially expressed genes between high- and low-risk patients revealed significant enrichment in immune-related pathways, including antigen presentation, cytokine–cytokine receptor interactions, and T cell activation. Single-cell RNA-seq further confirmed that low-risk patients exhibit more extensive immune cell interactions, particularly between CD8 + T cells, dendritic cells, and macrophages, which are critical for effective antitumor immunity. These results highlight the importance of cytokine-mediated immune regulation and suggest that targeting specific cytokine pathways could enhance anticancer immune responses.

While our study leverages large-scale transcriptomic datasets to explore the prognostic value of cytokines in breast cancer, several limitations should be noted. First, bulk RNA-seq inherently masks cellular heterogeneity by averaging gene expression across diverse cell populations. This limits our ability to resolve cell type-specific cytokine expression patterns and microenvironmental interactions. As highlighted by recent studies [46, 47], bulk RNA-seq data can be subject to both technical and biological confounders, including sampling bias, batch effects, and varying immune cell content, particularly in large consortia datasets like TCGA.

Second, although TCGA provides additional molecular layers, such as miRNA expression data, we did not incorporate miRNA-based biomarkers in this study. Our focus was to build a cytokine-centered classifier and risk model. However, integrating other regulatory elements such as miRNAs or circRNAs may enhance biomarker discovery. Other studies have emphasized the potential of noncoding RNAs to predict therapy resistance and improve treatment stratification [48]. Future studies incorporating multi-omics data may help refine the model and uncover additional regulatory mechanisms involved in cytokine signaling and therapeutic response.

Third, though we leveraged single-cell data to assess immune interactions, future studies integrating spatial transcriptomics could provide a more detailed view of cytokine-driven immune dynamics within the tumor microenvironment [49]. Incorporating such technologies in future work may help delineate the anatomical context of cytokine signaling and its role in shaping the tumor-immune interface. Beyond tissue-based analyses, advancements in liquid biopsy technologies also offer promising avenues for noninvasive monitoring of tumor biology. Techniques such as circulating tumor DNA (ctDNA) detection have demonstrated high clinical utility in various cancers, including breast cancer and neuroblastoma [50]. Similarly, next-generation sequencing combined with molecular barcode systems, enhances mutation detection sensitivity and specificity [51].

Moreover, methylation-based diagnostics have gained attention as potential early detection tools [52]. While our study is transcriptome-based, many cytokine-related pathways may also manifest alterations in circulating nucleic acid profiles or epigenetic states, raising the possibility of detecting cytokine-associated risk features through liquid biopsy approaches.

These innovations, when integrated with our cytokine-based classification, could assist in nodal management of breast cancer. Accurate assessment of immune activation and tumor burden is crucial for guiding surgical and radiotherapy decisions [53]. Future studies could evaluate whether cytokine-related biomarkers detected via noninvasive methods could inform regional treatment strategies or predict nodal involvement in the disease.

## 5. Conclusion

In conclusion, our study provides the first systematic characterization of cytokine gene expression in breast cancer, revealing distinct molecular subtypes with prognostic and therapeutic significance. By integrating transcriptomic profiling, immune landscape analysis, and clinical outcome prediction, we establish a cytokine-based classification system that reflects tumor immune heterogeneity and offers a novel framework for patient stratification. Furthermore, our cytokine-derived risk score demonstrates strong predictive value for both survival outcomes and treatment response, highlighting its potential as a clinically relevant biomarker. These findings underscore the critical role of cytokine signaling in breast cancer progression and immune regulation, suggesting that cytokine-based classification could guide personalized treatment strategies. Future studies incorporating multiomic approaches and prospective clinical validation will be essential to further refine this model and explore its potential for improving breast cancer immunotherapy and precision medicine.

## Data Availability

RNA sequencing (RNA-seq) and mutation data for breast cancer patients were obtained from the TCGA-BRCA cohort of The Cancer Genome Atlas (TCGA) database (https://www.cancer.gov/ccg/research/genome-sequencing/tcga). Single-cell RNA sequencing data can be found in a published work with GEO accession number GSE169246.
